# Raspberry aqueous extract ameliorates MAFLD in mice by regulating gut microbiota and purine metabolism

**DOI:** 10.3389/fnut.2026.1818086

**Published:** 2026-04-30

**Authors:** Yanyan Gao, Nian Liu, Huaxin Wei, Tianchi Sun, Fangying Xu, Lin Chen, Jiannan Qiu, Xiaobing Dou

**Affiliations:** 1 Zhejiang-Hong Kong Joint Laboratory of Liver and Spleen Simultaneous Treatment in Traditional Chinese Medicine, Zhejiang, China; 2 School of Life Sciences, Zhejiang Chinese Medical University, Hangzhou, Zhejiang, China; 3 School of Public Health, Zhejiang Chinese Medical University, Hangzhou, Zhejiang, China; 4 Lipid Metabolism Laboratory, Key Laboratory of State Administration of Traditional Chinese Medicine, Zhejiang, China; 5 Institute of Lipid Metabolism Zhejiang Chinese Medical University, Hangzhou, Zhejiang, China

**Keywords:** gut microbiota, inosine, metabolic-associated fatty liver disease, purine metabolism, *Rubus idaeus* L.

## Abstract

**Introduction:**

Metabolism-associated fatty liver disease (MAFLD) has emerged as a severe worldwide public health burden with insufficient available clinical therapeutic strategies, which underscores the urgent demand for safe, natural dietary interventions. Raspberry (*Rubus idaeus* L.), a typical food-medicine homologous fruit abundant in diverse bioactive components including anthocyanins, flavonoids and polysaccharides, possesses prominent nutritional and medicinal potential.

**Methods:**

In this study, raspberry aqueous extract (RE) was prepared to comprehensively investigate its ameliorative effects and underlying molecular mechanisms against MAFLD. MAFLD animal model was established in C57BL/6 mice via 12-week high-fat diet (HFD) feeding. From the 9th week, model mice were intragastrically administered with RE at doses of 1 g/kg/d and 2 g/kg/d for continuous intervention. Integrated multi-omics analyses including 16S rRNA microbial sequencing, serum/hepatic biochemical detection, histopathological examination, in vivo microbial colonization assay, and in vitro cellular and metabolomic experiments were performed to systematically clarify the regulatory mechanism.

**Results:**

RE treatment markedly improved the core pathological phenotypes of MAFLD mice, and significantly mitigated hepatic steatosis and hepatocellular injury. 16S rRNA sequencing demonstrated that RE remodeled the gut microbial dysbiosis, specifically elevating the abundance of beneficial genus *Ileibacterium* and suppressing pathogenic microbial taxa. Meanwhile, RE strengthened intestinal mucosal barrier integrity by upregulating tight junction protein expression, and activated hepatic purine metabolic reprogramming to boost the levels of critical metabolites including inosine and ADP. Spearman correlation analysis verified the significantly positive correlation between *Ileibacterium* abundance and hepatic inosine content, and both factors were closely correlated with the remission of MAFLD pathological indicators. In vivo colonization experiments further validated that *Ileibacterium* intervention alone remarkably alleviated hepatic lipid deposition and liver damage in MAFLD mice. In vitro strain metabolomics confirmed that *Ileibacterium* could directly biosynthesize and secrete inosine extracellularly. Furthermore, in vitro AML12 hepatocyte experiments revealed that 100 μM inosine remarkably relieved palmitic acid-induced lipotoxicity via reducing intracellular lipid overload, reactive oxygen species (ROS) accumulation and mitochondrial dysfunction, alongside modulating the expression of lipid metabolism, inflammatory and autophagy-related genes.

**Discussion:**

Collectively, our results elucidate that raspberry aqueous extract alleviates experimental MAFLD through the gut microbiota-purine metabolism-inosine regulatory axis, in which Ileibacterium and inosine act as the core synergistic mediators. This study provides solid preclinical experimental evidence for the development and application of raspberry as a promising functional food for the prevention and nutritional intervention of MAFLD.

## Graphical abstract

**Figure:**
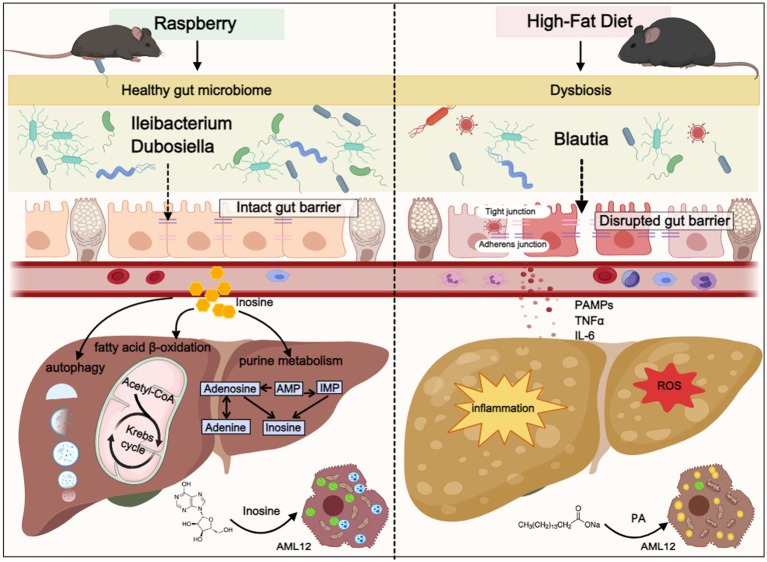

## 1 Introduction

Metabolism-associated fatty liver disease (MAFLD) has emerged as a global epidemic, with its prevalence soaring alongside the rising rates of obesity, type 2 diabetes, and metabolic syndrome. Currently affecting approximately one-third of the world’s population, MAFLD has surpassed viral hepatitis to become the leading cause of chronic liver disease worldwide (1, 2). Characterized by excessive hepatic lipid accumulation, MAFLD encompasses a spectrum of conditions ranging from simple steatosis to progressive metabolic-associated steatohepatitis (MASH), which can further develop into liver fibrosis, cirrhosis, and even hepatocellular carcinoma (3, 4). The pathogenesis of MAFLD is highly complex, involving the intricate interplay of genetic susceptibility, environmental factors, metabolic dysregulation, and gut-liver axis dysfunction (5, 6). Despite extensive research into its underlying mechanisms, effective therapeutic options remain limited, with lifestyle modifications being the mainstay of management. This critical gap highlights the urgent need to explore safe and natural interventions for the prevention and treatment of MAFLD.

Raspberry (*Rubus idaeus* L.), a member of the Rosaceae family, is a widely consumed fruit renowned for its rich nutritional profile and food-medicine homologous properties. It is abundant in vitamins, dietary fiber, minerals, and bioactive compounds such as anthocyanins, ellagic acid, flavonoids, and polysaccharides (7, 8). Accumulating evidence has demonstrated the multifaceted health benefits of raspberries, particularly in regulating metabolic homeostasis. Previous studies have shown that raspberry-derived bioactive components exert antioxidant and anti-inflammatory effects (9, 10), strengthen intestinal barrier function (11, 12), improve insulin sensitivity (13), and alleviate obesity-induced metabolic disorders (14). Raspberry ketone, a unique volatile compound in raspberries, has also been reported to reduce body weight and MAFLD in animal models (15, 16). However, the potential therapeutic effects of raspberry aqueous extract (RE) on MAFLD and the underlying molecular mechanisms, especially its interactions with the gut microbiota and metabolic pathways, remain insufficiently elucidated.

The gut microbiota, a complex community of microorganisms residing in the gastrointestinal tract, plays a pivotal role in maintaining host metabolic health and immune homeostasis. Mounting evidence indicates that gut microbiota dysbiosis is a key driver of MAFLD development (6, 17). High-fat diet (HFD) consumption, a major risk factor for MAFLD, disrupts the composition and diversity of the gut microbiota, leading to increased intestinal permeability, enhanced lipopolysaccharide (LPS) translocation, and chronic low-grade inflammation (18, 19). These alterations further impair hepatic lipid metabolism and glucose homeostasis, exacerbating hepatic steatosis and liver injury (20, 21). Additionally, metabolic pathways, particularly purine metabolism, are closely intertwined with MAFLD progression. Purine metabolites such as inosine and hypoxanthine have been shown to regulate lipid accumulation, insulin resistance, and energy metabolism (22–24), suggesting their potential as key mediators in MAFLD pathogenesis. However, the crosstalk between the gut microbiota and purine metabolism in the context of RE-induced MAFLD improvement remains unclear.

Given the therapeutic potential of raspberries and the critical role of the gut microbiota and metabolic pathways in MAFLD, the present study aimed to systematically investigate the effects of RE on HFD-induced MAFLD in mice. By integrating 16S rRNA gene sequencing for gut microbiota analysis and liquid chromatography–mass spectrometry (LC–MS)-based metabolomics, we sought to unravel the underlying mechanisms by which RE ameliorates MAFLD, with a specific focus on the modulation of gut microbiota composition and purine metabolism. The findings of this study will provide novel insights into the development of raspberry-based functional foods for MAFLD prevention and treatment, while shedding light on the intricate interactions between dietary interventions, gut microbiota, and metabolic pathways.

## 2 Materials and methods

### 2.1 Materials and reagents

The Raspberry was purchased from the Traditional Chinese Medicine Outpatient Department of Binjiang, Zhejiang Chinese Medical University (Origin: Zhejiang Province, China; Batch number: 1231201Y). A total of 200 g of raspberry was reflux-extracted twice with 2,000 mL of purified water at 100 °C for 1 h each time. After filtration, the combined filtrate was concentrated under reduced pressure and then freeze-dried to obtain 30 g of RE powder, with an extraction yield of 15%. HFD (batch number: D12492, fat content 60%) and normal diet (ND) were purchased from Trophic Animal Feed High-Tech Co., Ltd. (Nantong, China). Biochemical assay kits for alanine aminotransferase (ALT, batch number: C009-2-1), aspartate aminotransferase (AST, batch number: C010-2-1), total cholesterol (TC, batch number: A111-1-1), triglycerides (TG, batch number: A110-1-1), non-esterified fatty acids (NEFA, batch number: A042-2-1), and glycerol (batch number: F005-2-1) were provided by Nanjing Jiancheng Bioengineering Institute (Jiangsu, China). DAPI (batch number: P0131-25ML) was obtained from Beyotime Biotechnology (Shanghai, China). Free fatty acids (FFA) ELISA kit (batch number: SMK3062A) was supplied by Xiamen Senkos Technology Co., Ltd. (Xiamen, China), and Inosine ELISA kit (batch number: MM-45192 M1) was supplied by Beijing YaYuan Biotech Co., Ltd. (Beijing, China).

### 2.2 Anaerobic culture of *Ileibacterium valens*

*Ileibacterium valens* (ATCC No. TSD-63, purchased from Ningbo, China, catalog No. B260897) was revived and inoculated into culture medium (Ningbo, China, catalog No. KDM150) at a 4% (V/V) inoculum size, followed by incubation at 37 °C for 24 h. After cultivation, the bacterial suspension was centrifuged at 3,000 rpm for 10 min, and the resulting cell pellet was washed three times with pre-chilled sterile PBS at 4 °C. The cells were then resuspended in physiological saline and adjusted to a final concentration of 2 × 10^9^ colony-forming units (CFU) per 100 μL. To prepare heat-killed bacterial suspension, the aforementioned bacterial suspension was heated at 121 °C for 10 min. To confirm complete bacterial inactivation, plate-counting assays were performed to enumerate CFU in the suspensions before and after heat treatment, respectively. Bacterial supernatant, spent culture medium, and bacterial cell samples were separately collected for subsequent untargeted metabolomic analysis.

### 2.3 Animals

Male C57BL/6 mice (6–8 weeks old, weighing 20–22 g) were acquired from Shanghai SLAC Laboratory Animal Co., Ltd. (Shanghai, China), with a laboratory animal license number of SCXK (Hu) 2017–0005. Mice were housed in a specific pathogen-free (SPF) animal room with controlled temperature (22 ± 1 °C), humidity (55 ± 5%), and a 12 h light/dark cycle. They had free access to food and drinking water. After 1 week of acclimatization, mice were randomly divided into four groups (*n* = 8 per group): (a) normal diet group; (b) high-fat diet group; (c) RE low-dose treatment group; (d) RE high-dose treatment group. The normal group was given normal diet and the remaining three groups were given high-fat diet for 12 weeks. Groups (c) and (d) were given RE gavage treatment starting from the 9th week, at the same time group (a), (b) were given the same volume of saline by gavage, and at the end of 12 weeks fasting was done for 12 h, and mice were anesthetized with 1% pentobarbital sodium (60 mg/kg) then euthanized by cervical dislocation. Body weight of mice was measured weekly. Blood samples were collected from the inferior vena cava, centrifuged at 3,000 rpm for 15 min at 4 °C to separate plasma, which was stored at −80 °C. Liver, white adipose tissue (WAT), beige adipose tissue (Beige AT), brown adipose tissue (BAT), and intestinal tissues were quickly dissected, weighed, and part of the tissues were fixed in 4% paraformaldehyde for histological analysis, while the remaining tissues were stored at −80 °C for subsequent experiments. Colonic contents were collected and immediately frozen at −80 °C for the measurement of inosine concentration. Fresh fecal samples were collected before sacrifice and stored at −80 °C for gut microbiota analysis.

For the *Ileibacterium* colonization experiment, mice were randomly divided into four groups (*n* = 8 per group): (a) normal control group; (b) high-fat diet group; (c) *Ileibacterium valens*-treated group (*I.V*); (d) heat-killed *Ileibacterium valens* group (*I.V* + heat killing). Mice in the normal control group were fed a standard chow diet, whereas the remaining three groups were administered a high-fat diet continuously for 12 weeks. Starting from the 9th week, the *I.V* group received intragastric administration of *Ileibacterium* suspension at a dosage of 2 × 10^9^ CFU, while the *I.V* + heat killing group was given an equivalent inoculum (2 × 10^9^ CFU) of heat-killed *Ileibacterium* suspension *via* gavage.

### 2.4 Biochemical analysis

ALT, AST, TC, and TG were detected using an automatic biochemical analyzer (TK-40FR, Toshiba, Japan) following the manufacturer’s instructions. Liver tissue was homogenized in ice-cold physiological saline (1:9, w/v) using a tissue grinder, and the homogenate was centrifuged at 3,000 rpm for 10 min at 4 °C to collect the supernatant. The levels of TC and TG in liver tissue were measured using the corresponding assay kits. Feces samples were freeze-dried, ground into powder, and homogenized in ice-cold physiological saline (1:10, w/v). After centrifugation at 8,000 rpm for 20 min at 4 °C, the supernatant was collected to determine the levels of TG, NEFA, and glycerol using the respective kits.

### 2.5 Histological analysis

Liver, intestinal, and adipose tissues fixed in 4% paraformaldehyde were dehydrated through a graded ethanol series, embedded in paraffin, and sectioned into 4 μm slices. The sections were stained with hematoxylin and eosin (H&E) following standard protocols, and observed under a light microscope (Olympus BX53, Tokyo, Japan) to evaluate histological changes. For Oil Red O staining of liver tissue: Fresh liver tissue was embedded in Tissue-Tek OCT compound (Sakura Finetek, Tokyo, Japan), frozen at −80 °C, and sectioned into 6 μm slices. The slices were fixed in 4% paraformaldehyde for 10 min, rinsed with distilled water, stained with Oil Red O working solution for 15 min at 37 °C, differentiated with 60% isopropanol, counterstained with hematoxylin for 1 min, and observed under a light microscope to assess hepatic lipid accumulation.

### 2.6 Metabolomics analysis

A liver sample weighing 50 mg was placed in a 2 mL centrifuge tube, to which grinding beads and 400 μL of an extraction solution (methanol:water = 4:1, v:v) containing 0.02 mg/mL of the internal standard L-2-chlorophenylalanine were added for metabolite extraction. The sample solution was subjected to grinding using a cold tissue grinder for 6 min at −10 °C and 50 Hz, followed by low-temperature ultrasonic extraction for 30 min at 5 °C and 40 kHz. Subsequently, the samples were allowed to stand at −20 °C for 30 min, after which they were centrifuged for 15 min at 4 °C and 13,000 × g. The supernatant was then transferred to an injection vial equipped with an insert tube for subsequent analysis. Data analysis was conducted using the Majorbio Cloud platform, accessible at https://cloud.majorbio.com.

### 2.7 Gut microbiota analysis

Total DNA was extracted from fecal samples using the E. Z. N. A.^®^ soil DNA kit (Omega Bio-tek, Norcross, GA, United States) according to the manual. Based on the quantity and quality of the extracted DNA, samples were selected for subsequent sequencing. The 16S rRNA gene V3-V4 variable region was amplified by PCR using the upstream primer 338F (5′-ACTCCTACGGGAGGCAGCAG-3′) and the downstream primer 806R (5′-GGACTACHVGGGTWTCTAAT-3′). The PCR products were recovered using a 2% agarose gel, purified with the DNA Gel Recovery Purification Kit (PCR Clean-Up Kit, Yunhua, China), and quantified using Qubit 4.0 (Thermo Fisher Scientific, United States). Sequencing was performed using the Illumina Nextseq2000 platform (Shanghai Majorbio Bio-pharm Technology Co., Ltd.). All data analysis was conducted on the Majorbio Bio-cloud platform (https://cloud.majorbio.com).

### 2.8 FFA and inosine level analysis

Plasma samples were thawed on ice and mixed thoroughly. Colonic contents were diluted with pre-cooled saline at a mass-to-volume ratio of 1:9 (m/v), homogenized on ice, and centrifuged at 12,000 rpm for 10 min at 4 °C. The supernatant was collected for analysis. The levels of FFA and inosine were determined using ELISA kits according to the manufacturer’s instructions. FFA was measured only in plasma, while inosine was measured in both plasma and colonic supernatant. Sample concentrations were calculated based on optical density (OD) values and standard curves. The inosine concentration in colonic contents was expressed in μg/mg and calculated from the supernatant concentration, dilution factor, supernatant volume, and tissue weight.

### 2.9 Immunofluorescence staining

The intestinal tissue paraffin sections were subjected to immunofluorescent staining using primary antibodies against ZO-1, Occludin, and Claudin-1(all diluted at 1:400). Multi-rAb CoraLite® Plus 594 (1:400) or CoraLite® Plus 488 (1:400) was used as the secondary antibody, and the cell nuclei were stained with DAPI. The images were captured using an Olympus VS120 microscope (Olympus, Tokyo, Japan). The antibodies employed are detailed in Supplementary Table S1.

### 2.10 Quantitative RT-PCR analysis

Liver total mRNA was extracted using the FastPure® Cell/Tissue Total RNA Isolation Kit (Vazyme, Nanjing, China), and cDNA was obtained by reverse transcription kit (Vazyme, Nanjing, China) as the template of real-time PCR reaction. The Ct values were automatically calculated by the computer after qPCR, and calculations and statistics were performed using the 2^(−ΔΔct)^ method. Details of the primers synthesized by Tsingke Biotech Co. Ltd. (Beijing, China) are in Supplementary Table S2.

### 2.11 Cell culture

AML12 mouse hepatocytes (ATCC) were cultured in DMEM/F12 (319-085-CL, Wisent, China) supplemented with 10% FBS (12003C, Sigma-Aldrich, United States), 1 × ITS (10 μg/mL insulin, 5 μg/mL transferrin, 5 ng/mL selenium; I3146, Sigma-Aldrich, United States), 40 ng/mL dexamethasone (D4902, Sigma-Aldrich, United States), and 1% streptomycin/penicillin/gentamicin (BL141A, Biosharp, China). Cells were maintained at 37 °C with 5% CO₂. For treatment, cells were exposed to 0.5 mM PA (P9767-10G, Sigma-Aldrich, United States) to induce steatosis, with inosine treatment (25 μM, 50 μM, 100 μM, 200 μM, 400 μM). A non-treated control group was included.

### 2.12 Cell viability

AML12 hepatocytes were seeded in the plates in the previous day. Cells were treated at 50–70% confluence. Cell viability was assessed after the procedure was finished using the Cell Counting Kit-8 (CCK8) assay (Beyotime, China) according to the manufacturer’s instructions.

### 2.13 Bodipy, mtROS, and JC-1 staining

AML12 hepatocytes were incubated with Bodipy 493/503 dye (1 μM in the culture medium) for lipid detection, MitoSOX Red dye (1 μM in the culture medium) for mtROS detection, Hoechst dye (1 μM in the culture medium) for nuclear staining and JC-1 dye (1 μM in the culture medium) for mitochondrial membrane potential assessment at 37 °C for 30 min. Subsequently, images were acquired using a fluorescence microscope (ZEISS, Oberkochen, Germany), employing an optimally configured combination of filters to ensure precise visualization and quantification of the fluorescent signal. Using Image J (Fiji version 1.8.0), Bodipy and mtROS fluorescence quantifications were performed by measuring the mean fluorescence intensity after background subtraction, followed by normalization to the control samples to ensure accurate and reproducible analysis of lipid accumulation and reactive oxygen species.

### 2.14 Data analysis

Data were expressed as mean ± standard deviation (SD). For comparisons among multiple groups, one-way ANOVA followed by Tukey’s *post hoc* test was applied if both assumptions were met; otherwise, the non-parametric Kruskal-Wallis test followed by Dunn’s *post hoc* test was used. For comparisons between two groups, the Student’s *t*-test was used for normally distributed data, and the Mann–Whitney U test was used for data that violated the normality assumption, both conducted in GraphPad Prism 9 (GraphPad Software, San Diego, CA, United States).

## 3 Results

### 3.1 RE improves liver injury of MAFLD mice

**Figure 1:**
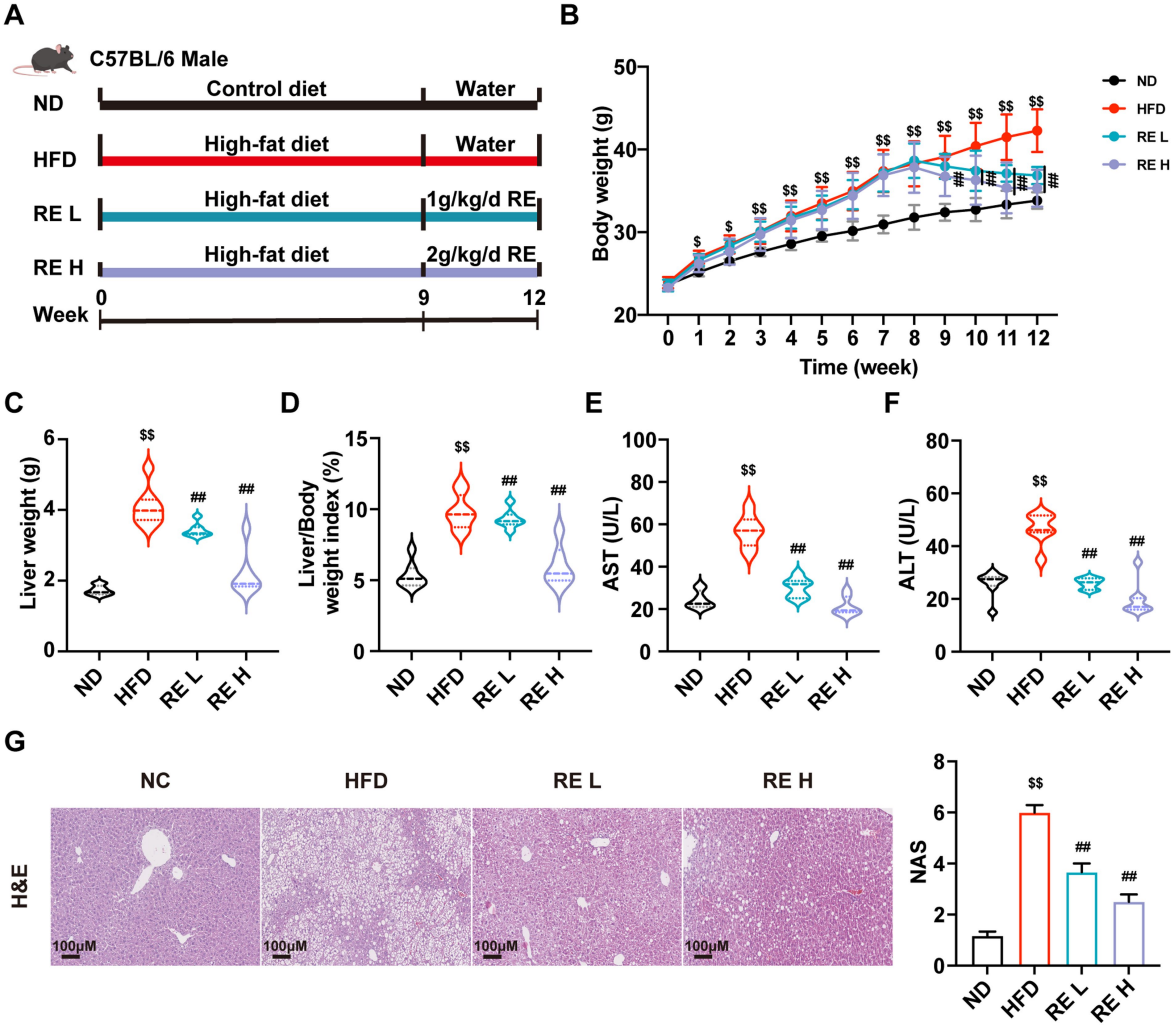
RE improves liver injury of MAFLD mice. **(A)** Experimental flow chart. **(B)** Weight growth curves (weekly). **(C,D)** Liver weight and liver/body weight ratio of mice in each group. **(E,F)** Plasma AST and ALT levels of mice. **(G)** H&E staining of mouse liver histological sections. Data are expressed as mean ± SD (*n* = 8). ^$^*p* < 0.05, ^$$^*p* < 0.01 compared with ND group. ^#^*p* < 0.05, ^##^*p* < 0.01 compared with HFD group.

To establish the MAFLD model, mice were fed a high-fat diet for 12 weeks, and RE intervention was administered *via* gavage from week 9 (Figure 1A). The body weight of HFD-fed mice was significantly higher than that of ND mice from week 1, while RE treatment (both low and high doses) gradually reduced body weight from week 9 (Figure 1B). At the end of the 12-week experiment, compared with the HFD group, RE-treated mice showed dose-dependent decreases in liver weight and liver-to-body weight ratio (Figures 1C,D). Biochemical analysis revealed that plasma levels of AST and ALT, key markers of liver injury, were significantly elevated in the HFD group but effectively lowered by RE intervention (Figures 1E,F). Histological observation *via* H&E staining showed severe hepatic steatosis in HFD-fed mice, characterized by disordered hepatocyte arrangement and excessive lipid accumulation, while RE treatment notably alleviated these pathological changes (Figure 1G). These results confirm that RE exerts a protective effect against MAFLD-induced liver damage.

### 3.2 RE alleviates hepatic lipid accumulation in MAFLD mice

**Figure 2:**
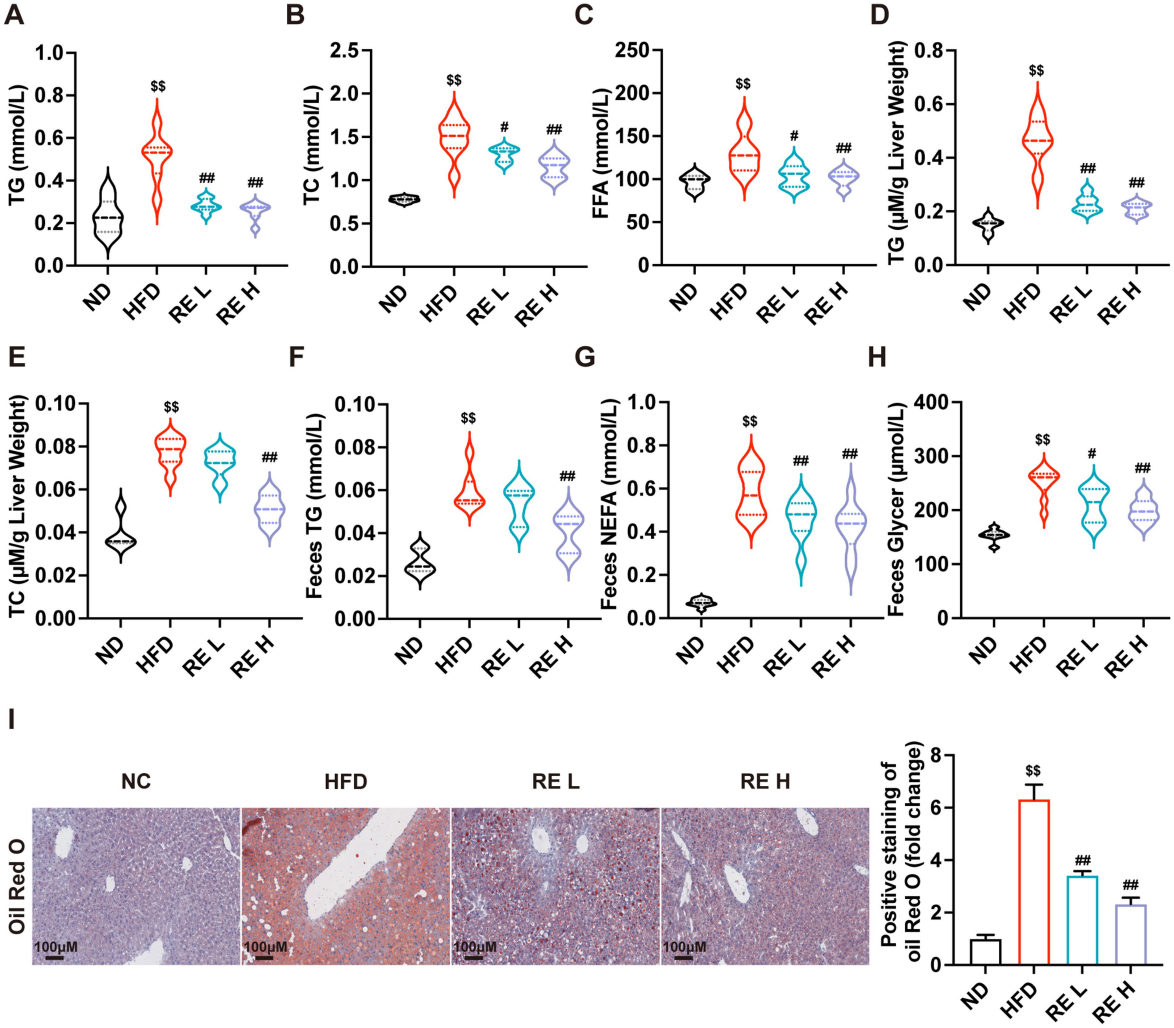
RE alleviates hepatic lipid accumulation in MAFLD mice. **(A–E)** TG, TC, FFA levels in plasma and TG, TC levels in liver tissue. **(F–H)** The levels of TG, NEFA and Glycerol in feces of mice. **(I)** Oil red O staining of mouse liver histological sections. Data are expressed as mean ± SD (*n* = 8). ^$^*p* < 0.05, ^$$^*p* < 0.01 compared with ND group. ^#^*p* < 0.05, ^##^*p* < 0.01 compared with HFD group.

To evaluate the effect of RE on lipid metabolism disorders, we measured the levels of TG and TC in plasma and liver tissues. The results showed that HFD feeding significantly increased TG, TC, and FFA levels in plasma, as well as TG and TC levels in the liver, while RE intervention (especially at the high dose) significantly reversed these elevations (Figures 2A–E). Additionally, the levels of TG, NEFA, and glycerol in feces were significantly higher in the HFD group than in the ND group, and these indices were significantly improved after RE treatment (Figures 2F–H), suggesting that RE can regulate intestinal fat metabolism and ameliorate systemic lipid disorders in MAFLD mice. Oil Red O staining further demonstrated that HFD-fed mice had a large number of lipid droplets in liver tissue, whereas RE treatment reduced the number and size of lipid droplets, indicating improved hepatic steatosis (Figure 2I).

### 3.3 RE reduces fat accumulation and adipocyte hypertrophy in HFD mice

**Figure 3:**
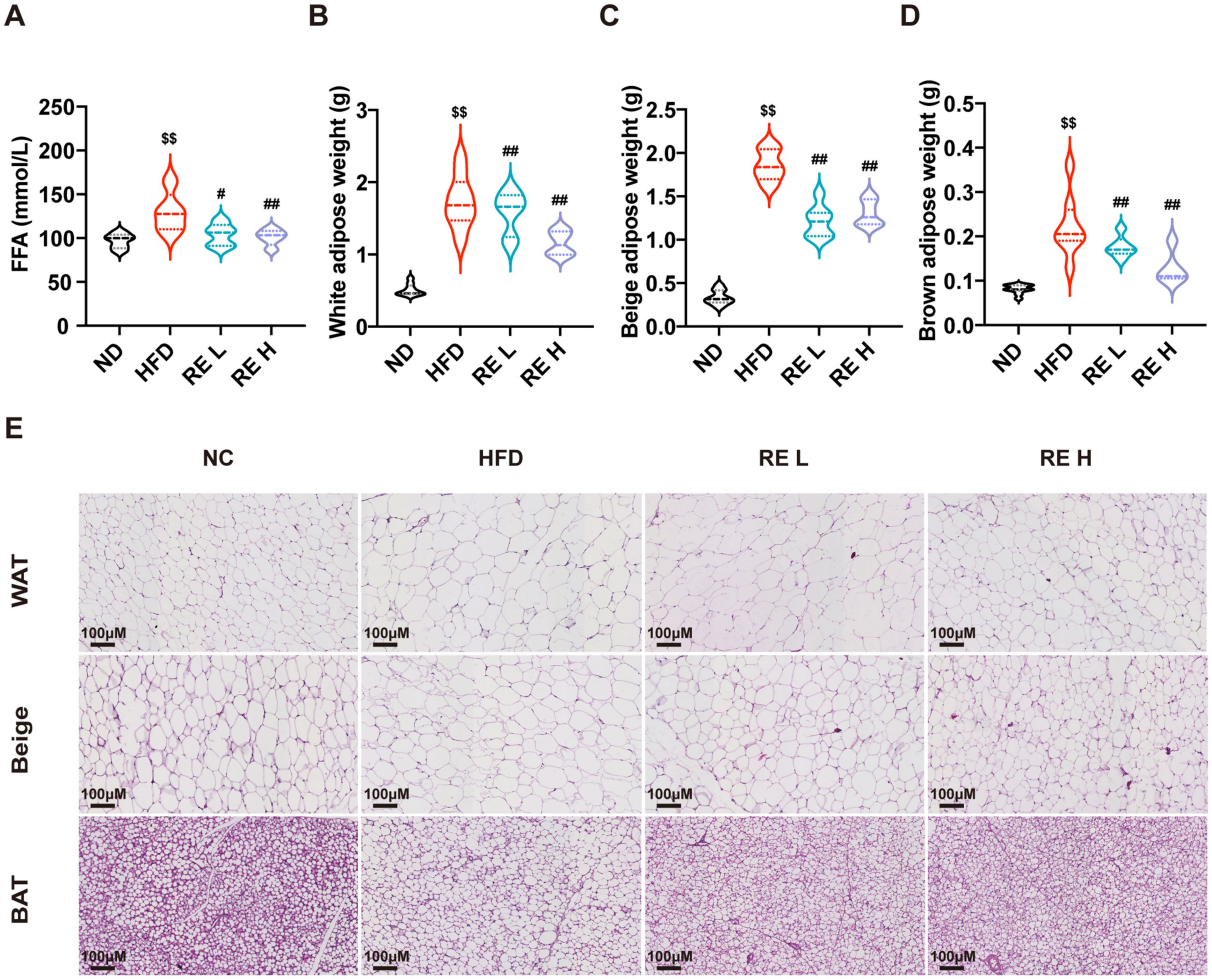
RE reduces fat accumulation and adipocyte hypertrophy in HFD mice. **(A)** Plasma FFA level in mice. **(B–D)** The weight of white, beige and brown adipose tissue. **(E)** H&E staining of mouse (White, Beige, and BAT) adipose histological sections. Data are expressed as mean ± SD (*n* = 8). ^$^*p* < 0.05, ^$$^*p* < 0.01 compared with ND group. ^#^*p* < 0.05, ^##^*p* < 0.01 compared with HFD group.

Plasma FFA levels were significantly increased in HFD-fed mice, and RE intervention effectively reversed this elevation (Figure 3A). Moreover, HFD feeding led to significant increases in the weights of WAT, Beige AT, and BAT, while RE treatment significantly reduced the weights of these adipose tissues (Figures 3B–D). H&E staining of adipose tissue sections showed that adipocytes in the HFD group were larger in size, irregular in shape, and loosely arranged, whereas RE-treated mice had smaller adipocytes with a more compact and regular arrangement, and an increased number of adipocytes per field of view (Figure 3E). These results indicate that RE can inhibit adipose tissue hyperplasia and improve adipocyte morphology in MAFLD mice.

### 3.4 RE regulates gut microbiota diversity and composition in MAFLD mice

**Figure 4:**
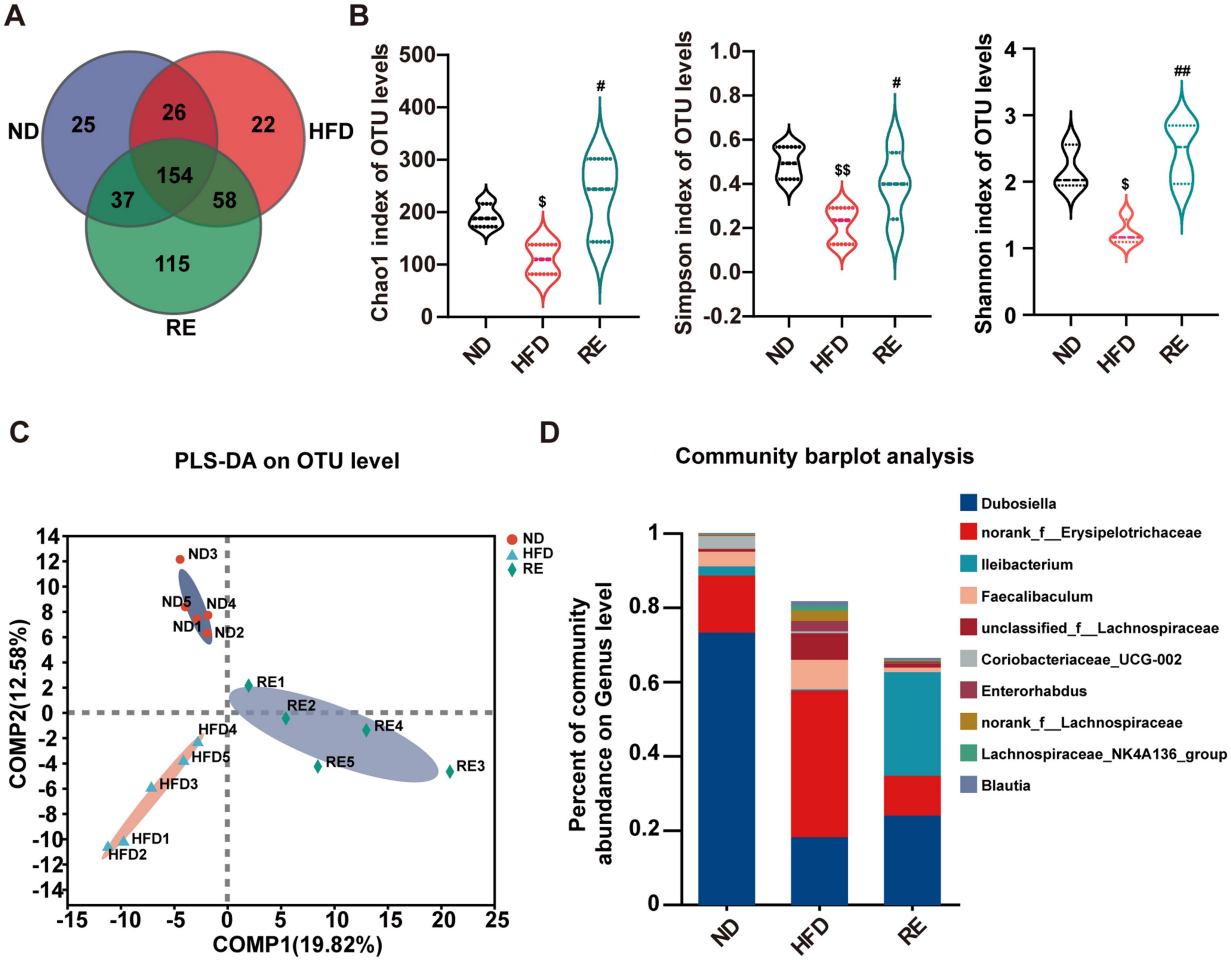
RE regulates gut microbiota diversity and composition in MAFLD mice. **(A)** Venn diagram based on OTU level. **(B)** The Alpha analysis of gut microbiota in three groups of mice. **(C)** PLS-DA score map of gut microbiota. **(D)** The barplot showed the relative abundances of main genus bacteria. Data are expressed as mean ± SD (*n* = 5). $*p* < 0.05, $$*p* < 0.01 compared with ND group. ^#^*p* < 0.05, ^##^*p* < 0.01 compared with HFD group.

16S rRNA sequencing of fecal was performed to analyze the effect of RE on gut microbiota. Venn diagram analysis at the operational taxonomic units (OTU) level showed that a total of 436 OTUs were identified among the ND, HFD, and RE groups, with 154 overlapping OTUs among the ND, HFD and RE groups, suggesting that RE modulates gut microbiota composition (Figure 4A). Alpha diversity analysis revealed that compared with the ND group, the HFD group exhibited decreased Chao1, Shannon, and Simpson indices (indicating reduced microbial richness and evenness), while RE intervention reversed these changes, restoring gut microbiota alpha diversity (Figure 4B). Partial least squares discriminant analysis (PLS-DA) showed clear separation of gut microbiota composition among the three groups, and RE intervention altered the gut microbiota structure of HFD-fed mice (Figure 4C). At the genus level, HFD feeding reduced the abundance of *Dubosiella* and *Ileibacterium*, while increasing the abundance of *Enterorhabdus*, *norank_f__Erysipelotrichaceae*, *Faecalibaculum*, *unclassified_f__Lachnospiraceae* and *Blautia*. All these changes were significantly reversed by RE treatment (Figure 4D).

### 3.5 *Ileibacterium* is closely correlated with MAFLD phenotypes

**Figure 5:**
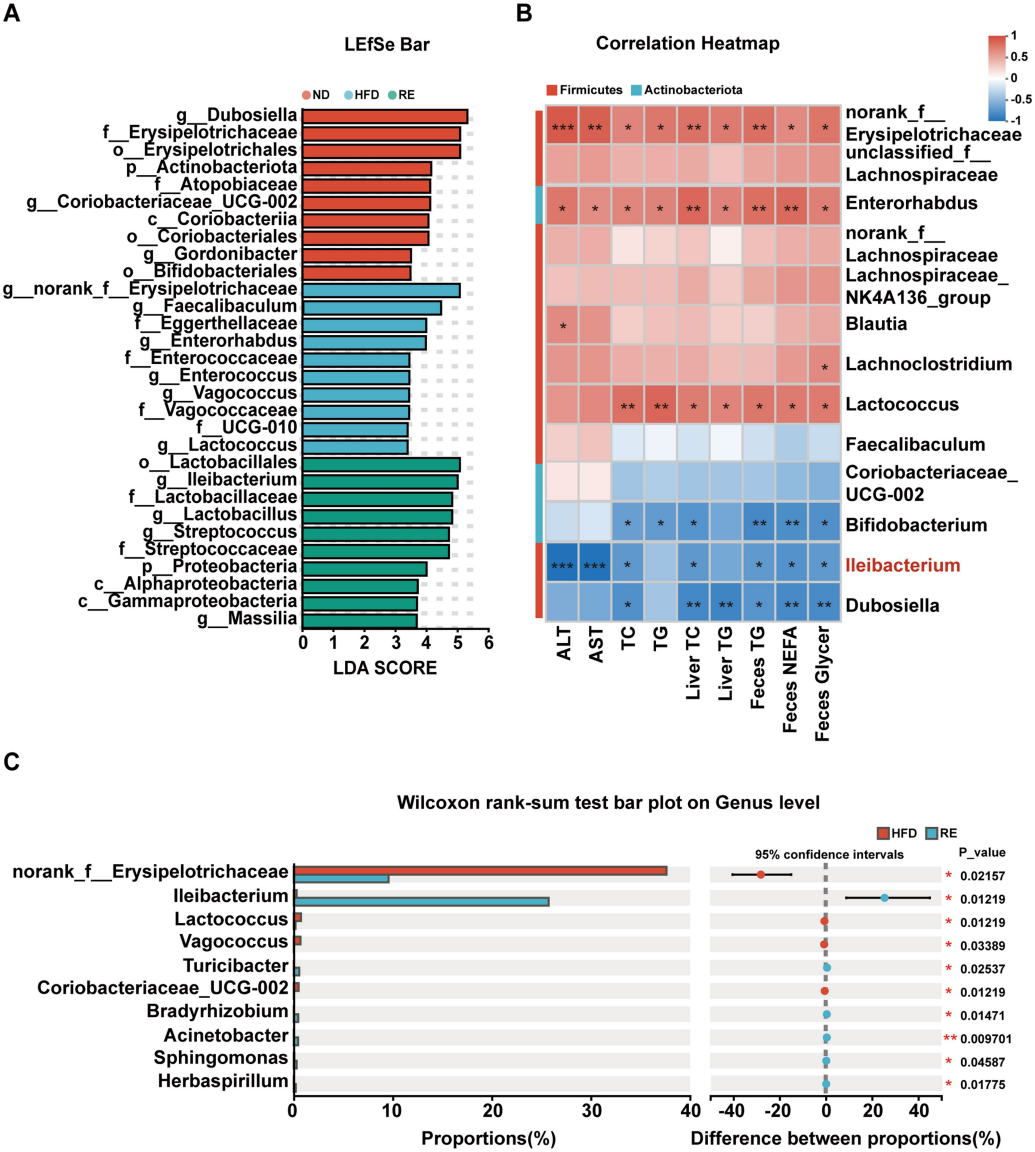
*Ileibacterium* is closely correlated with MAFLD phenotypes. **(A)** Linear discriminant analysis (LDA ≥ 2.0) scores derived from LEfSe Analysis. **(B)** The correlation between the changed genera responding to MAFLD-related index. **(C)** Statistical analysis of the top 10 bacterial species with different bacterial abundance between HFD and RE group. *p < 0.05, **p < 0.01, ***p < 0.001. Data are expressed as mean ± SD (*n* = 5).

LEfSe analysis showed that the HFD group was enriched in *g_norank_f_Erysipelotrichaceae* and *g_Faecalibaculum*, while the RE group was enriched in *o_Lactobacillales* and *g_Ileibacterium* (Figure 5A). Statistical analysis confirmed that compared with the HFD group, RE treatment significantly increased the abundance of *Ileibacterium* and decreased the abundance of *norank-f-Erysipelotrichaceae* (Figure 5C). Spearman correlation analysis further revealed that *Ileibacterium* abundance was strongly correlated with MAFLD-related indicators, showing negative correlations with plasma ALT, AST, TC, liver TC, fecal TG, fecal NEFA, and fecal glycerol levels (Figure 5B). Meanwhile, *in vivo* intervention experiments targeting *Ileibacterium* further verified its direct regulatory role, as viable *Ileibacterium* administration significantly reduced body weight, liver weight, and serum levels of ALT, AST, TC, and TG in MAFLD mice, whereas heat-inactivated *Ileibacterium* treatment markedly attenuated these protective effects. These findings indicate that *Ileibacterium* may be a key microbial target for RE to improve MAFLD (Supplementary Figures S3A–F).

### 3.6 RE enhances intestinal barrier function in HFD-fed mice

**Figure 6:**
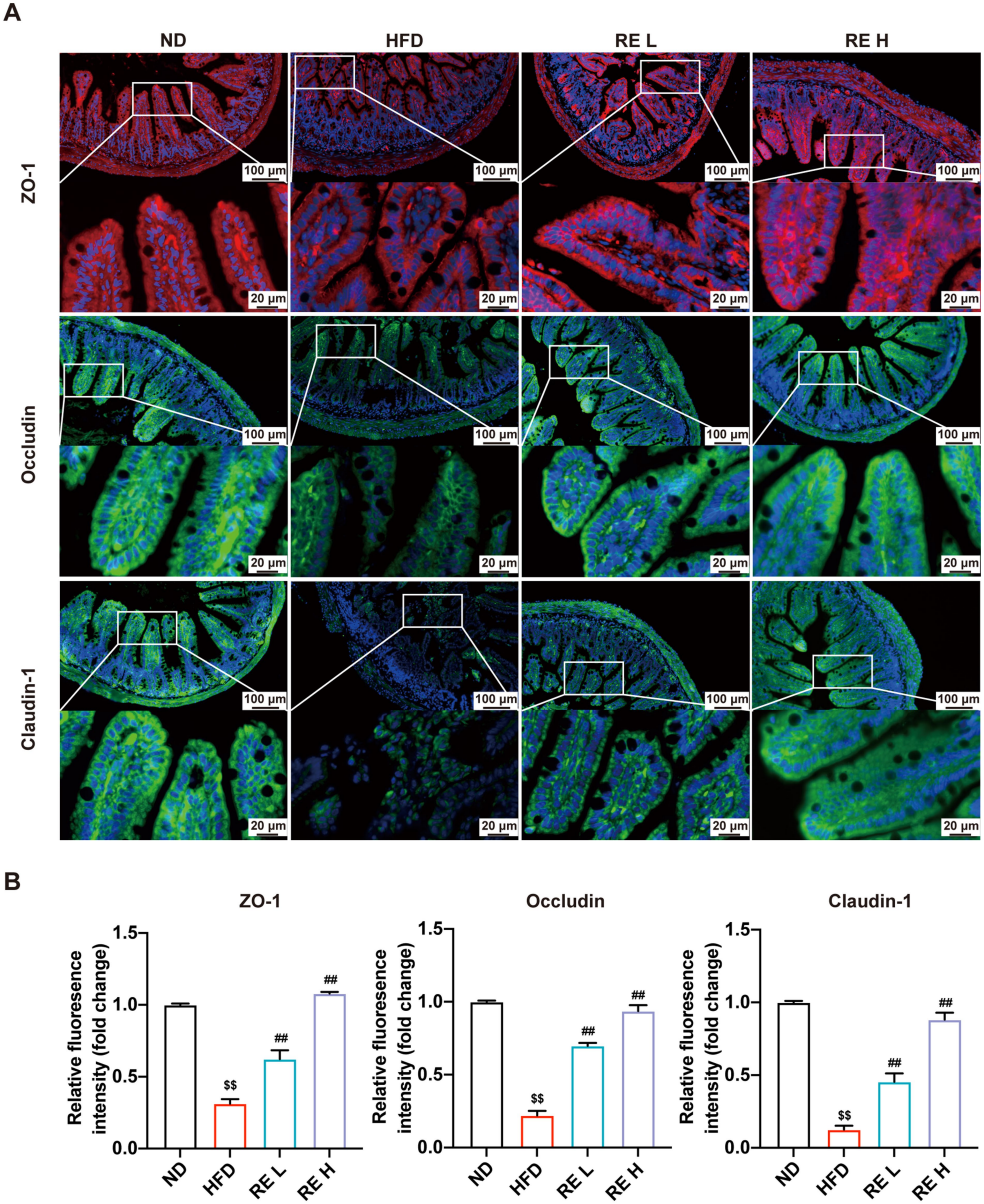
RE enhances intestinal barrier function in HFD-fed mice. **(A)** Immunofluorescence staining of mouse intestine for ZO-1 (red), Occludin (green), and Claudin-1 (green). **(B)** Relative quantification of barrier protein fluorescence intensity. Data are expressed as mean ± SD (*n* = 3). ^$^*p* < 0.05, ^$$^*p* < 0.01 compared with ND group. ^#^*p* < 0.05, ^##^*p* < 0.01 compared with HFD group.

Immunofluorescence staining was used to detect the expression of tight junction (TJ) proteins ZO-1, Occludin, and Claudin-1 in intestinal tissue. The results showed that HFD feeding significantly reduced the expression of ZO-1, Occludin, and Claudin-1, indicating impaired intestinal barrier function. In contrast, RE intervention (especially high dose) upregulated the expression of these TJ proteins, and the expression levels in the RE high-dose group were close to those in the ND group (Figure 6). These findings suggest that RE can alleviate HFD-induced intestinal barrier damage and enhance intestinal barrier integrity.

### 3.7 RE regulates hepatic purine metabolism in MAFLD mice

**Figure 7:**
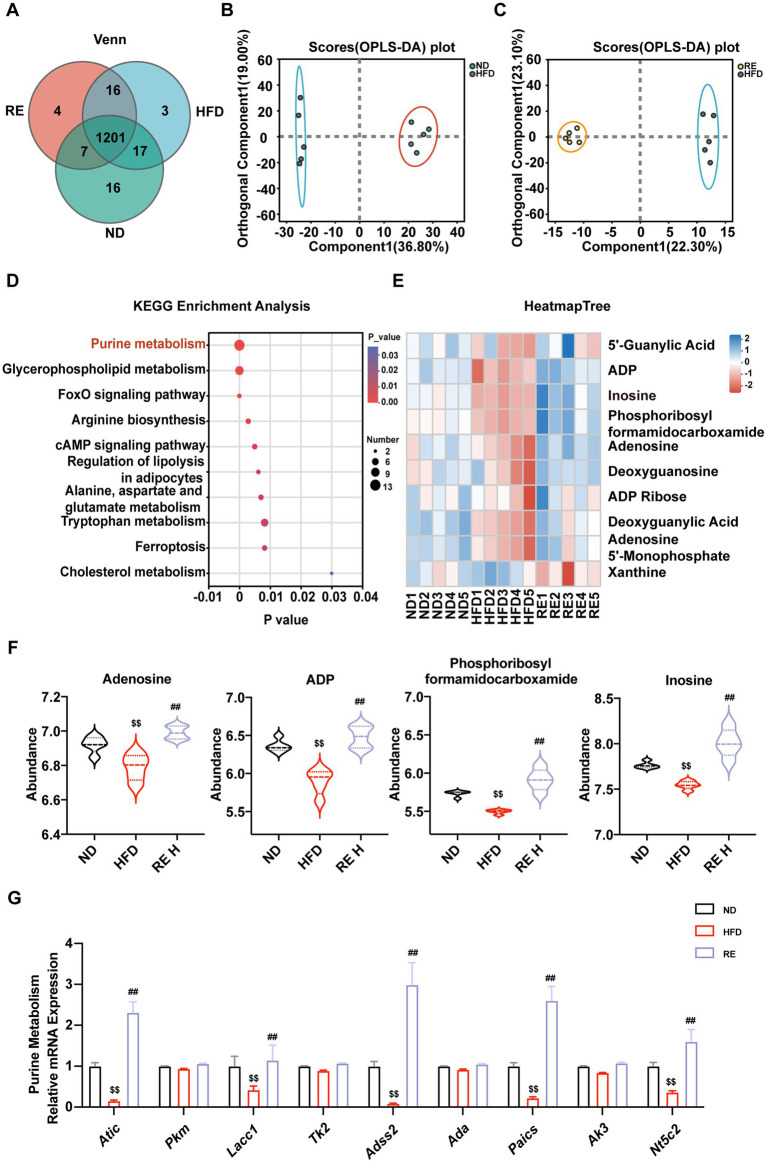
RE regulates hepatic purine metabolism in MAFLD mice. **(A)** Venn diagram of liver tissue Metabolomics. **(B,C)** OPLS-DA score map of liver tissue metabolomics. **(D)** Analysis of Metabolomics KEGG enrichment signaling pathway. **(E)** The heatmap of Purine signaling pathway after RE treatment. **(F)** Relative abundance statistics of inosine, ADP and phosphoribosyl formamidocarboxamide. **(G)** Gene expression related to purine metabolism pathway in liver tissue. Data are expressed as mean ± SD (*n* = 5). ^$^*p* < 0.05, ^$$^*p* < 0.01 compared with ND group. ^#^*p* < 0.05, ^##^*p* < 0.01 compared with HFD group.

Untargeted metabolomics analysis of liver tissue identified a total of 1,264 metabolites among the three groups, with 1,201 shared metabolites (Figure 7A). Orthogonal partial least squares discriminant analysis (OPLS-DA) showed clear separation between the ND and HFD groups, as well as between the HFD and RE groups, indicating that RE significantly alters hepatic metabolic profiles (Figures 7B,C). KEGG pathway enrichment analysis revealed that RE intervention significantly regulated multiple metabolic pathways, with purine metabolism being the most prominently enriched (Figure 7D). Specifically, RE treatment significantly increased the levels of purine metabolism-related metabolites such as inosine, ADP, and phosphoribosyl formamidocarboxamide (Figures 7E,F). Quantitative RT-PCR analysis further confirmed that RE intervention upregulated the expression of purine metabolism-related genes, including *Nt5c2*, *Lacc1*, *Adss2*, *Paics*, and *Atic* (Figure 7G), indicating that purine metabolism is a key pathway through which RE regulates hepatic metabolism in MAFLD mice.

### 3.8 Correlation analysis between metabolites and MAFLD phenotype in mice

**Figure 8:**
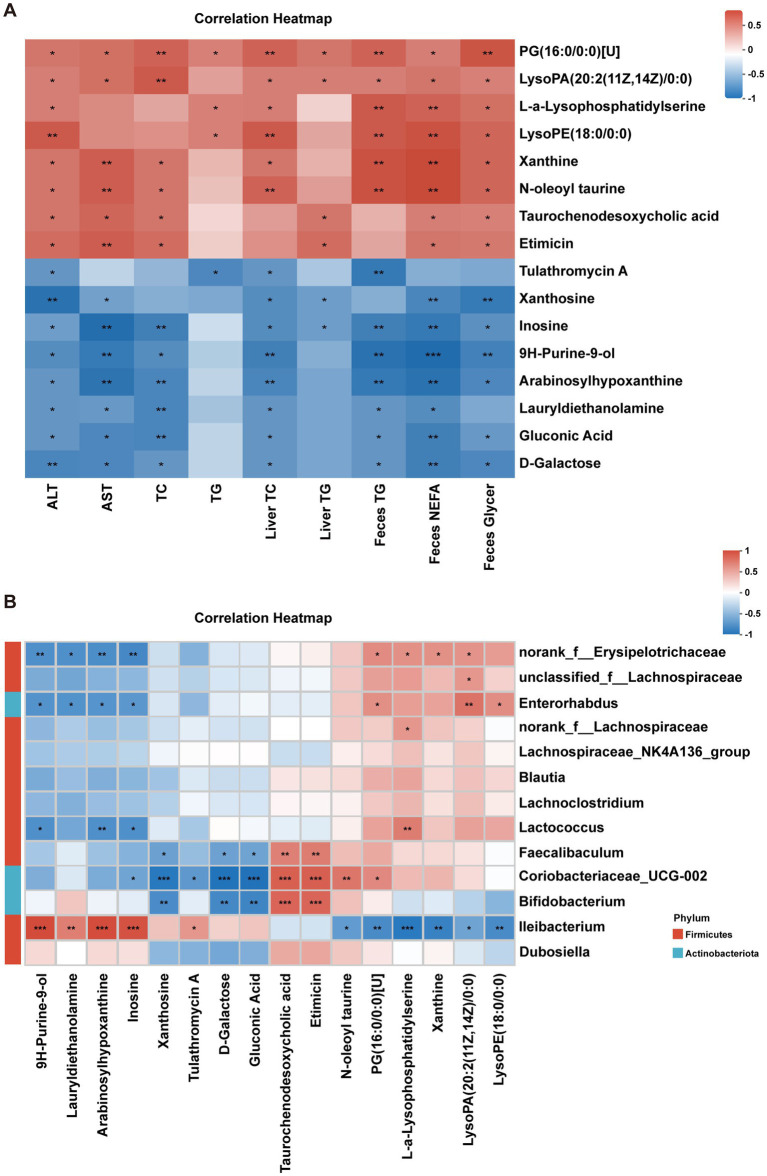
Correlation analysis between metabolites and MAFLD phenotype in mice. **(A)** The correlation between the changed metabolite responding to MAFLD-related index. **(B)** The correlation between the significantly changed genera and metabolites. *p < 0.05, **p < 0.01, ***p < 0.001.

Spearman correlation analysis between differential metabolites and MAFLD-related indicators showed that inosine was strongly negatively correlated with plasma AST, ALT, TC, liver TC, liver TG, fecal TG, fecal NEFA, and fecal glycerol levels, suggesting that inosine may be a key metabolite mediating the beneficial effects of RE on MAFLD (Figure 8A). Additionally, correlation analysis between significantly altered gut microbiota genera and differential metabolites revealed that *Ileibacterium* was strongly positively correlated with inosine, Arabinosylhypoxanthine, and 9H-Purine-9-ol, and negatively correlated with L-α-Lysophosphatidylserine and xanthine (Figure 8B), ELISA quantification showed that the change trends of inosine levels in colonic contents and plasma were consistent with the abundance of *Ileibacterium* and hepatic inosine levels (Supplementary Figures S1A,B). Further *in vitro* metabolomic analysis of *Ileibacterium* cultures confirmed that this bacterium could directly synthesize and secrete inosine into the culture supernatant (Supplementary Figures S4A–C). These results indicate a functional crosstalk between *Ileibacterium* and inosine, whereby *Ileibacterium* may contribute to RE-mediated MAFLD amelioration *via* inosine synthesis and secretion.

### 3.9 Inosine alleviates palmitic acid-induced damage in AML12 cells

**Figure 9:**
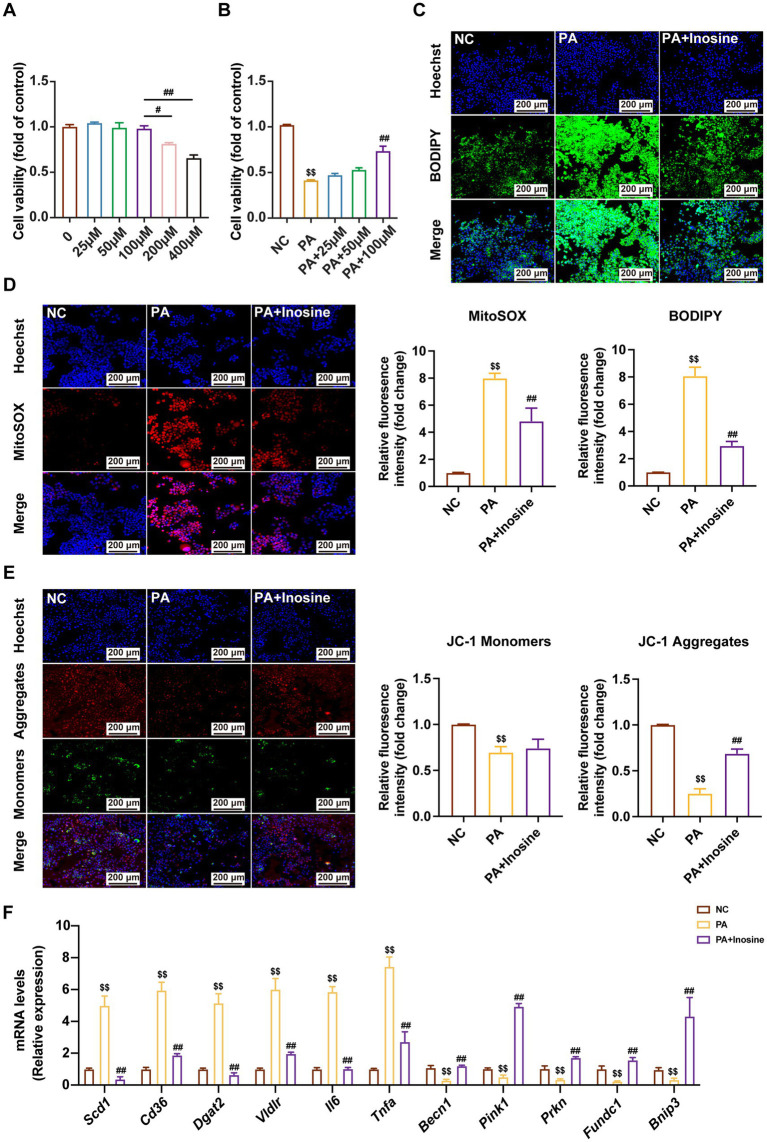
Inosine alleviates palmitic acid (PA)-induced damage in AML12 cells. **(A,B)** Cell viability. **(C)** The effect of inosine (100 μM) intervention on Bodipy staining in PA-induced AML12 cells. **(D)** mtROS was detected and quantified by MtSOX *in vitro*, Scale bar = 200 μm. **(E)** The effect of inosine (100 μM) intervention on JC-1 staining in PA-induced AML12 cells. **(F)** The relative expression of genes in lipid accumulation, pro-inflammatory factors, and autophagy. Data are expressed as mean ± SD (*n* = 4). ^$^*p* < 0.05, ^$$^*p* < 0.01 compared with NC group. ^#^*p* < 0.05, ^##^*p* < 0.01 compared with PA group.

To examine the protective effects of inosine against palmitic acid (PA)-induced hepatocellular damage, AML12 cells were exposed to PA stimulation with or without inosine pretreatment, followed by an analysis of various cellular functional indicators. As illustrated in Figures 9A,B, inosine at concentrations of 100 μM and below demonstrated no cytotoxicity, and treatment with 100 μM inosine significantly ameliorated the damage induced by PA in AML12 cells. To further confirm the optimal concentration, we supplemented functional experiments with additional inosine concentrations (25 μM and 50 μM) alongside the original 100 μM group. *In vitro*, PA exposure led to increased lipid accumulation, elevated reactive oxygen species (ROS) levels, and mitochondrial dysfunction in AML12 cells; these effects were mitigated by inosine treatment in a dose-dependent manner, with the most pronounced effects observed at 100 μM (Figures 9C–E; Supplementary Figures S2A,B). Additionally, quantitative reverse transcription PCR (qRT-PCR) results indicated that inosine treatment significantly suppressed the PA-induced upregulation of genes associated with lipid accumulation (such as *Scd1, Cd36, Dgat2*, and *Vldlr*) and pro-inflammatory factors (*Il-6*, *Tnfα*, and *Il-1β*), while enhancing the expression of autophagy-related genes (including *Becn1*, *Pink1*, *Prkn*, *Fundc1*, and *Bnip3*) in a dose-dependent manner, with the strongest regulatory effects at 100 μM (Figure 9F; Supplementary Figure S2C).

## 4 Discussion

MAFLD has evolved into a global metabolic crisis, with its pathogenesis intricately linked to gut-liver axis dysfunction, metabolic reprogramming, and chronic low-grade inflammation (25). The lack of safe and effective therapeutic strategies has prompted intensive exploration of natural products with food-medicine homologous properties. Our study systematically demonstrates that RE ameliorates HFD-induced MAFLD in mice, and further validates that inosine, a key metabolite regulated by RE, exerts direct protective effects on PA-induced lipid metabolism disorders and inflammatory injury in AML12 cells. These findings collectively unravel a novel mechanism by which RE improves MAFLD through the “gut microbiota-inosine-hepatic metabolism” axis, providing compelling evidence for the development of raspberry-based functional foods against metabolic liver diseases.

The phenotypic improvements induced by RE in MAFLD mice are multifaceted and clinically relevant (26). HFD feeding triggers excessive weight gain, hepatic hypertrophy, and elevated plasma transaminases (AST, ALT)-classic hallmarks of liver injury and metabolic dysfunction (27–29). RE intervention dose-dependently reversed these abnormalities, accompanied by reduced hepatic steatosis and improved hepatocyte morphology. These observations align with previous reports on the metabolic benefits of raspberry-derived bioactive compounds, such as anthocyanins and polysaccharides, in alleviating obesity and hepatic lipid accumulation (30, 31). However, our study extends beyond these surface-level effects, uncovering the underlying systemic regulatory network: RE not only targets the liver but also modulates intestinal lipid absorption (evidenced by reduced fecal TG, NEFA, and glycerol levels) and adipose tissue remodeling (characterized by smaller adipocytes and reduced adipose weight). This multi-tissue regulatory effect suggests that RE acts as a holistic metabolic modulator, addressing the systemic nature of MAFLD rather than isolated hepatic pathology.

Gut microbiota dysbiosis is a well-recognized driver of MAFLD, as it disrupts metabolic balance, impairs intestinal barrier function, and promotes chronic inflammation (32). Our 16S rRNA sequencing results revealed that HFD feeding induced significant changes in gut microbiota composition and diversity: compared to the ND group, the HFD group showed reduced alpha diversity (lower Chao1, Shannon, and Simpson indices), which is consistent with findings in human MAFLD patients who also exhibit decreased gut microbial alpha diversity (33). RE intervention reversed these HFD-induced changes, restoring microbial richness and evenness. At the genus level, HFD feeding reduced the abundance of beneficial bacteria such as *Dubosiella* and *Ileibacterium*-genera whose depletion has been linked to colitis, diabetes, and obesity (34–36), while raising the levels of *Enterorhabdus* (positively correlated with inflammation levels) (37), and *Blautia* (linked to intestinal dysfunction) (38). Importantly, RE treatment normalized the abundance of these genera, suggesting that it modulates gut microbiota composition to restore intestinal homeostasis. Spearman correlation analysis further identified *Ileibacterium* as a key microbial genus: its abundance was strongly correlated with MAFLD-related indicators (e.g., plasma ALT/AST, hepatic TC, fecal lipid metabolites), aligning with previous studies showing that *Ileibacterium* abundance is negatively correlated with blood glucose levels (39). Monocolonization experiments *in vivo* further verified the hepatoprotective effect of *Ileibacterium*. This highlights *Ileibacterium* as a potential mediator of RE’s anti-MAFLD effects.

Metabolomic profiling and *in vitro* validation collectively pinpoint inosine as a pivotal effector molecule in RE’s therapeutic pathway. Hepatic metabolomics analysis revealed that purine metabolism is the most significantly enriched pathway after RE intervention, with inosine, ADP, and phosphoribosyl formamidocarboxamide being the key upregulated metabolites. Correlation analysis further demonstrated that inosine levels are strongly negatively associated with MAFLD-related pathological indicators, while positively correlated with the abundance of *Ileibacterium*. Metabolomic analysis of *Ileibacterium* cultured *in vitro* further confirmed that this bacterium can synthesize and secrete inosine. This suggests a potential causal link: RE modulates gut microbiota to promote inosine production, which in turn ameliorates hepatic metabolic disorders. The direct protective role of inosine was confirmed *in vitro*: exogenous inosine supplementation alleviated PA-induced lipid accumulation in AML12 cells and suppressed the expression of pro-inflammatory cytokines. This is consistent with recent studies showing that inosine regulates lipid metabolism by promoting energy expenditure and inhibiting lipogenesis, and exerts anti-inflammatory effects by modulating adenosine receptor signaling (40, 41). Our study bridges the gap between *in vivo* gut microbiota-metabolite correlations and *in vitro* functional validation, establishing inosine as a key mediator that translates RE-induced gut microbiota changes into hepatic protective effects.

The interplay between gut microbiota, inosine, and intestinal barrier function adds another layer of complexity to the mechanism. HFD-induced gut dysbiosis impairs intestinal barrier integrity by downregulating tight junction proteins, leading to increased intestinal permeability and metabolic endotoxemia (42, 43). RE intervention not only restores tight junction protein expression but also enriches *Ileibacterium*, which may directly or indirectly promote inosine production. Inosine, in turn, could further reinforce intestinal barrier function or be transported to the liver *via* the portal vein to exert its lipid-lowering and anti-inflammatory effects. This “gut microbiota-inosine-barrier-liver” feedback loop may represent a key regulatory network by which RE maintains metabolic homeostasis. Additionally, RE’s regulation of purine metabolism is supported at the transcriptional level: genes involved in purine synthesis and metabolism (*Nt5c2, Lacc1, Adss2, Paics*) are upregulated after RE intervention, indicating that RE modulates purine metabolism through both microbial and hepatic intrinsic pathways.

The *in vitro* findings on inosine’s protective effects in AML12 cells further strengthen the clinical relevance of our study. Palmitic acid, a major saturated fatty acid in the Western diet, is a key inducer of hepatic lipotoxicity and inflammation-mimicking the pathological environment of MAFLD (44, 45). Our results show that inosine supplementation directly reduces PA-induced lipid droplet accumulation and inhibits the expression of pro-inflammatory mediators, suggesting that inosine could serve as a potential therapeutic agent for MAFLD. This complements the *in vivo* observations, confirming that inosine is not merely a correlative metabolite but an active effector molecule. Together, these data propose a clear mechanistic chain: RE reshapes the gut microbiota to increase *Ileibacterium* abundance, which promotes inosine production; inosine then acts on hepatocytes to alleviate lipid metabolism disorders and inflammation, ultimately ameliorating MAFLD.

Several limitations of this study should be acknowledged. First, while we demonstrate a positive correlation between *Ileibacterium* and inosine, this cross-organ correlation is indirect and affected by multiple physiological and confounding factors, which cannot represent a direct regulatory relationship. Future studies could further explore the specific molecular mechanisms underlying the regulation of inosine synthesis and secretion by *Ileibacterium*. Second, the specific molecular targets of inosine in hepatocytes (e.g., adenosine receptors, metabolic enzymes) require further investigation to fully elucidate its signaling pathways. Third, the bioactive components in RE responsible for gut microbiota modulation and inosine upregulation (e.g., polyphenols, polysaccharides) need to be identified and purified to enhance the translational potential of our findings.

In conclusion, our study uncovers a novel mechanism by which raspberry aqueous extract ameliorates MAFLD through the coordinated regulation of gut microbiota, purine metabolism, and hepatic function. The key insights are threefold: (1) RE reshapes gut microbiota composition, particularly enriching *Ileibacterium*; (2) RE upregulates hepatic inosine levels *via* both gut microbiota modulation and direct regulation of purine metabolism genes; (3) Inosine exerts direct protective effects against lipid accumulation and inflammation in hepatocytes. These findings not only provide a deeper understanding of the interaction between dietary natural products, gut microbiota, and metabolic health but also offer a promising strategy for the prevention and management of MAFLD using raspberry-derived products or inosine-related interventions. Future clinical trials are warranted to validate these preclinical findings and explore the therapeutic potential of RE and inosine in human MAFLD.

This study demonstrates that RE ameliorates HFD-induced MAFLD in mice. The core mechanism involves the regulation of the “gut microbiota-purine metabolism” axis: RE reshapes gut microbiota composition, notably enriching the beneficial bacterium *Ileibacterium*, and upregulates hepatic purine metabolism to increase key metabolites such as inosine. *In vitro* experiments further confirm that inosine directly alleviates palmitic acid-induced lipid accumulation and inflammatory injury in AML12 cells. The positive correlation between *Ileibacterium* and inosine highlights their synergistic role in mediating RE’s therapeutic effects. These findings identify RE as a promising natural intervention for MAFLD and provide novel insights into the gut microbiota-metabolite-liver crosstalk, laying a foundation for the development of functional foods targeting metabolic liver diseases.

## Data Availability

The datasets presented in this study can be found in online repositories. The names of the repository/repositories and accession number(s) can be found in the article/Supplementary material.
