# Supplementary material for: Raspberry aqueous extract ameliorates MAFLD in mice by regulating gut microbiota and purine metabolism
Source: Front Nutr. 2026 Apr 30;13:1818086. doi: 10.3389/fnut.2026.1818086 (PMC13171365; doi:10.3389/fnut.2026.1818086)
Supplement: Supplementary file 1 [file Supplementary_file_1.docx]

**Raspberry aqueous extract ameliorates MAFLD in mice by regulating gut microbiota and purine metabolism**

Yanyan Gao^1,2,*^, Nian Liu^1,2,*^, Tianchi Sun^3*^, Fangying Xu^1,2^, Lin Chen^1,2,4,5^, Huaxin Wei^1,2,#^, Jiannan Qiu^1, 2,4,5, #^, Xiaobing Dou ^1, 2,4,5, #^

***Affiliations***

*^1^ Zhejiang-Hong Kong Joint Laboratory of Liver and Spleen Simultaneous Treatment in Traditional Chinese Medicine, Zhejiang, PR China, 310053*

*^2^ School of Life Sciences, Zhejiang Chinese Medical University, Hangzhou, Zhejiang, PR China, 310053*

*^3^ School of Public Health, Zhejiang Chinese Medical University, Hangzhou, Zhejiang, PR China, 310053*

*^4^ Lipid Metabolism Laboratory, Key Laboratory of State Administration of Traditional Chinese Medicine, Zhejiang, PR China, 310053*

*^5^ Institute of Lipid Metabolism Zhejiang Chinese Medical University, Hangzhou, Zhejiang, PR China, 310053*

*These authors contribute equally to this paper.

^#^ To whom correspondence should be addressed E-mail: [7394822@qq.com](mailto:7394822@qq.com) (Q. Liu) and [xbdou77@163.com](mailto:xbdou77@163.com) (X. Dou).

^#^ Corresponding author (J. Qiu) at: School of Life Science, Zhejiang Chinese Medical University, No. 548, Binwen Road, Binjiang District, Hangzhou, Zhejiang, 310053, China. Office Phone number: (0571) 8661 3598

^#^ Corresponding author (X. Dou) at: School of Life Science, Zhejiang Chinese Medical University, No. 548, Binwen Road, Binjiang District, Hangzhou, Zhejiang, 310053, China. Office Phone number: (0571) 8661 3598

**
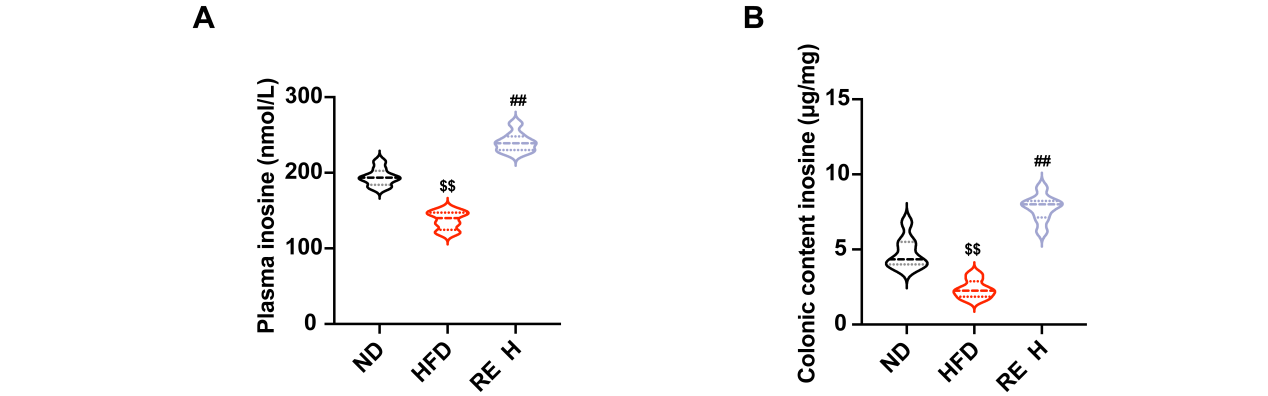
**

**Figure S1. RE regulates hepatic purine metabolism in MAFLD mice.** (A) Plasma inosine levels. (B) Colonic content inosine levels. Data are expressed as mean ± SD (n = 8). ^$^*p* < 0.05, ^$$^*p* < 0.01 compared with ND group. ^#^*p* < 0.05, ^##^*p* < 0.01 compared with HFD group.


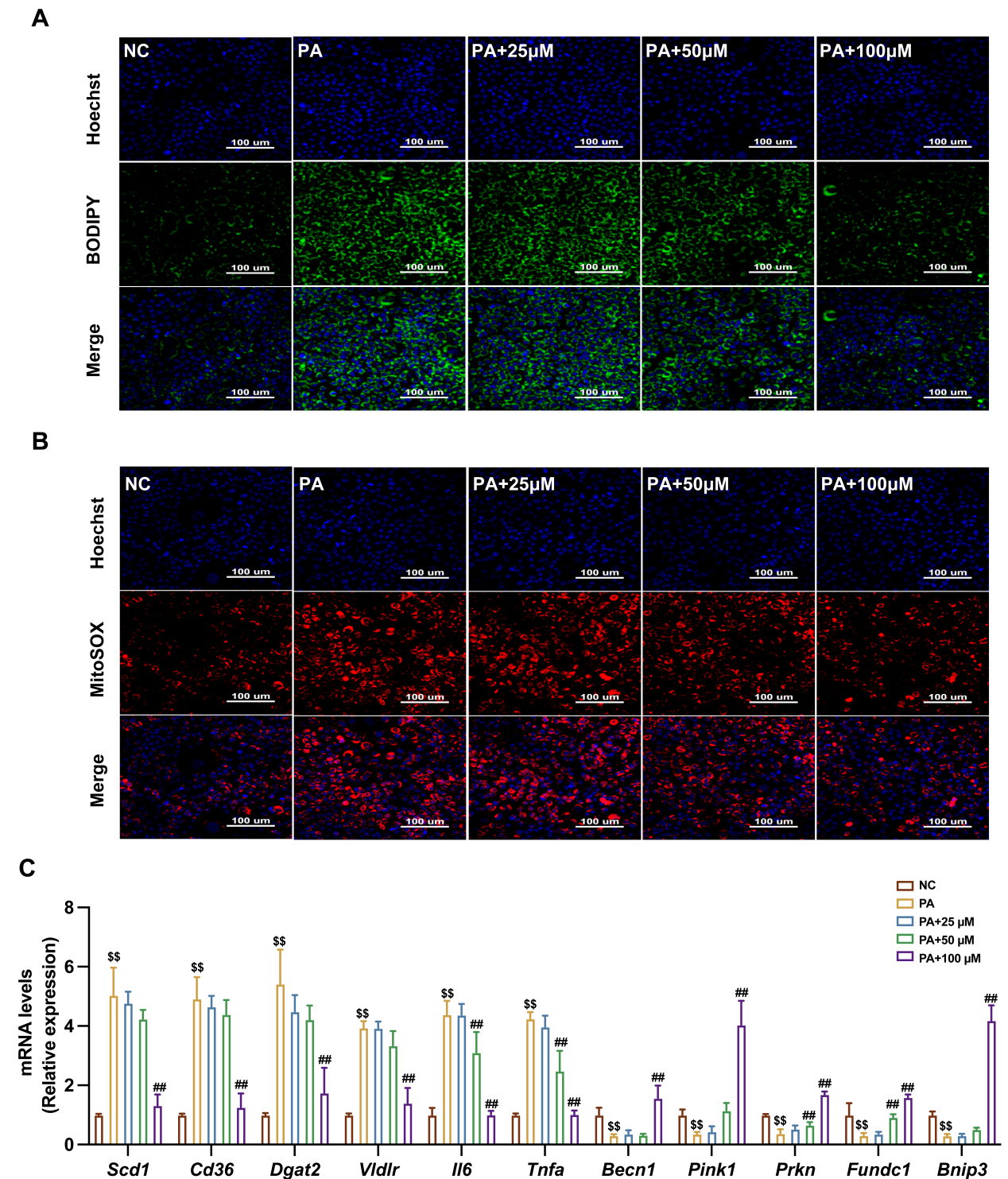


**Figure S2. 100 μM Inosine alleviates palmitic acid (PA)-induced damage in AML12 cells.** (A) The effects of inosine at different concentrations on Bodipy staining in PA-induced AML12 cells. (B) The effects of inosine at different concentrations on MtSOX staining in PA-induced AML12 cells. Scale bar = 100 μm. (C) The relative expression of genes in lipid accumulation, pro‑inflammatory factors, and autophagy. Data are expressed as mean ± SD (n = 4).^$^*p* < 0.05, ^$$^*p* < 0.01 compared with NC group. ^#^*p* < 0.05, ^##^*p* < 0.01 compared with PA group.

**Figure S3. *Ileibacterium* ameliorates liver injury and lipid accumulation in MAFLD mice.** (A) Body weight of mice in each group. (B) Liver weight of mice in each group. (C&D) Plasma AST and ALT levels of mice. (E&F) Plasma TC and TG levels of mice. Data are expressed as mean ± SD (n = 8). ND: normal diet, HFD: high-fat diet, *I.V*: high-fat diet + *Ileibacterium*, *I.V* + heat killing: high-fat diet + heat-killed *Ileibacterium*. ^*^*p* < 0.05, ^**^*p* < 0.01.

**Figure S4. *Ileibacterium* regulates purine metabolism in MAFLD mice.** (A)Venn Diagram. (B) PLS-DA Score Plot. (C) Volcano plot . Data are expressed as mean ± SD (n = 3). JT: bacterial pellet group, PYJ: medium control group, SQ: supernatant group.
