# Supplementary material for: Raspberry aqueous extract ameliorates MAFLD in mice by regulating gut microbiota and purine metabolism
Source: Front Nutr. 2026 Apr 30;13:1818086. doi: 10.3389/fnut.2026.1818086 (PMC13171365; doi:10.3389/fnut.2026.1818086)
Supplement: Supplementary file 2 [file Table_1.docx]

**Aqueous extract of raspberry ameliorates hepatic lipotoxicity in MAFLD mice by regulating gut microbiota and purine metabolism**

Yanyan Gao^1,2,*^, Nian Liu^1,2,*^, Huaxin Wei^1,2,*^, Tianchi Sun^3,*^, Fangying Xu^1,2^, Lin Chen^1,2,4,5^, Jiannan Qiu^1, 2,4,5, #^, Xiaobing Dou ^1, 2,4,5, #^

***Affiliations***

*^1^ Zhejiang-Hong Kong Joint Laboratory of Liver and Spleen Simultaneous Treatment in Traditional Chinese Medicine, Zhejiang, PR China, 310053*

*^2^ School of Life Sciences, Zhejiang Chinese Medical University, Hangzhou, Zhejiang, PR China, 310053*

*^3^ School of Public Health, Zhejiang Chinese Medical University, Hangzhou, Zhejiang, PR China, 310053*

*^4^ Lipid Metabolism Laboratory, Key Laboratory of State Administration of Traditional Chinese Medicine, Zhejiang, PR China, 310053*

*^5^ Institute of Lipid Metabolism Zhejiang Chinese Medical University, Hangzhou, Zhejiang, PR China, 310053*

*These authors contribute equally to this paper.

^#^ To whom correspondence should be addressed E-mail: qjntcm@163.com (J. Qiu) and [xbdou77@163.com](mailto:xbdou77@163.com) (X. Dou).

^#^ Corresponding author (X. Dou) at: School of Life Science, Zhejiang Chinese Medical University, No. 548, Binwen Road, Binjiang District, Hangzhou, Zhejiang, 310053, China. Office Phone number: (0571) 8661 3598

^#^ Corresponding author (J. Qiu) at: School of Life Science, Zhejiang Chinese Medical University, No. 548, Binwen Road, Binjiang District, Hangzhou, Zhejiang, 310053, China. Office Phone number: (0571) 8661 3598

**Table S1**. Characterization of compounds in RE by UPLC-Q-TOF-MS.

| **NO.** | **tR(min)** | **Identification** | **Formula** | **Detected *m/z*** | **ppm** | **Main MS/MS fragments** |
| --- | --- | --- | --- | --- | --- | --- |
| 1 | 2.24 | 2,3-(S)-hexahydroxydiphenoyl-D-glucose | C20H18O14 | 481.0619[M-H]^-^ | -1.0 | 300.9986, 275.0188 |
| 2 | 8.64 | Strictinin | C27H22O18 | 633.0734[M-H]^-^ | 0.1 | 481.0661, 300.9993, 275.0197 |
| 3 | 11.53 | Brevifolin carboxylic acid | C13H8O8 | 291.0155[M-H]^-^ | 3.0 | 247.0247,191.0343,190.0263,145.0290 |
|  |  |  | C13H8O8 | 293.0296[M+H]^+^ | 1.4 | 219.0291, 191.0341, 163.0392 |
| 4 | 13.84 | 3,5,9-trihydroxy-7,8dihydrocyclopenta[g]chromene-2,6-dione | C12H8O6 | 249.0395[M+H]^+^ | 0.5 | 207.0285, 179.0336, 151.0389 |
| 5 | 15.22 | Ellagic acid 4-O-α-L-arabinofuranoside | C19H14O12 | 433.0414[M-H]^-^ | 0.3 | 299.9910 |
| 6 | 15.91 | Ellagic acid | C14H6O8 | 303.0141[M+H]^+^ | 1.8 | 257.0081, 201.0184 |
|  |  |  | C14H6O8 | 301.0002[M-H]^-^ | 4.0 | 299.9919,283.9966,229.0138,185.0243 |
| 7 | 16.92 | Isoquercitrin | C21H20O12 | 465.1026[M+H]^+^ | -0.4 | 303.0492 |
|  |  |  | C21H20O12 | 463.0883[M-H]^-^ | 0.2 | 301.0368,300.0284,271.0258,255.0308 |
| 8 | 16.92 | Quercetin | C15H10O7 | 303.0501[M+H]^+^ | 0.5 | 229.0498, 153.0187, 137.0241 |
| 9 | 16.92 | Hyperoside | C21H20O12 | 465.1027[M+H]^+^ | -0.1 | 303.0492 |
|  |  |  | C21H20O12 | 463.0883[M-H]^-^ | 0.2 | 301.0368,300.0284,271.0258,255.0308 |
| 10 | 18.15 | Kaempferol | C15H10O6 | 287.0552[M+H]^+^ | 0.6 | 153.0181 |
| 11 | 18.16 | Kaempferol-3-O-rutinoside | C27H30O15 | 595.1659[M+H]^+^ | 0.3 | 287.0553 |
|  |  |  | C27H30O15 | 593.1518[M-H]^-^ | 1.0 | 285.0404, 284.0324, 255.0297 |
| 12 | 18.16 | Nicotiflorin | C27H30O15 | 595.1659[M+H]^+^ | 0.3 | 287.0553 |
| 13 | 18.17 | Kaempferol-3-rutinoside | C27H30O15 | 593.1520[M-H]^-^ | 1.4 | 285.0404, 284.0324, 255.0297 |
| 14 | 18.17 | Nicotiflorin | C27H30O15 | 593.1518[M-H]^-^ | 1.0 | 285.0404, 284.0324, 255.0297 |
| 15 | 18.90 | Astragalin | C21H20O11 | 447.0934[M-H]^-^ | 0.2 | 285.0408, 284.0333, 255.0300, 227.0348 |
|  |  |  | C21H20O11 | 449.1082[M+H]^+^ | 0.7 | 287.0548 |
| 16 | 18.90 | Quercitrin | C21H20O11 | 447.0934[M-H]^-^ | 0.2 | 285.0408,284.0333,255.0300,227.0348 |
|  |  |  | C21H20O11 | 449.1082[M+H]^+^ | 0.7 | 287.0548 |
| 17 | 19.12 | 18-O-β-D-xylopyranosyl-18S-hydroxydihydroprotolichesterinate 21-O-β-D-glucopyranoside | C32H56O14 | 709.3662[M+FA-H]^-^ | 1.4 | 663.3672 |
|  |  |  |  | 682.4010[M+NH4]^+^ | 0.2 | 287.0551, 269.2267 |
| 18 | 24.73 | Aromadendrin | C15H12O6 | 287.0567[M-H]^-^ | 2.0 | 272.0320, 243.0288, 216.0422 |
| 19 | 25.08 | Goshonoside-F5 isomer1 | C32H54O13 | 691.3557[M-H]^-^ | 1.5 | 645.3570,499.2949,485.1118,319.1917 |
|  |  |  |  | 664.3897[M+NH4]^+^ | -0.9 | 287.2370,269.2264,229.1949,219.1744, 201.1635 |
| 20 | 25.61 | Cis-tiliroside | C30H26O13 | 595.1442[M+H]^+^ | -0.7 | 291.0858, 287.0548, 147.0444 |
|  |  |  | C30H26O13 | 593.1304[M-H]^-^ | 0.5 | 285.0408,284.0334,255.0295,277.0347 |
| 21 | 25.62 | Tiliroside | C30H26O13 | 595.1442[M+H]^+^ | -0.7 | 291.0858, 287.0548, 147.0444 |
|  |  |  | C30H26O13 | 593.1304[M-H]^-^ | 0.5 | 285.0408, 284.0334, 255.0295, 277.0347 |
| 22 | 25.85 | Goshonoside-F5 isomer2 | C32H54O13 | 681.3272[M+Cl]^-^ | 2.0 | 645.3560 |
|  |  |  |  | 664.3898[M+NH4]^+^ | -0.7 | 287.2364, 269.2261, 229.1947 |
| 23 | 26.08 | Goshonoside-F5 | C32H54O13 | 691.3557[M-H]^-^ | 1.5 | 645.3561, 483.2988, 179.0554 |
|  |  |  |  | 664.3893[M+NH4]^+^ | -1.5 | 287.2367, 269.2265, 229.1948 |
| 24 | 26.63 | Paniculoside IV | C26H42O9 | 497.2762[M-H]^-^ | 1.2 | 479.2685, 317.2126 |
| 25 | 26.91 | Goshonoside-F1 | C26H44O8 | 529.3021[M+FA-H]^-^ | 0.5 | 484.7527, 483.2998, 179.0555, |
|  |  |  |  | 485.3108[M+H]^+^ | -0.2 | 287.2367, 229.1949, 199.1491 |
| 26 | 27.04 | Goshonoside-F2 | C26H44O8 | 529.3022[M+FA-H]^-^ | 0.7 | 484.4176, 483.2986, 321.2437, 161.0447, 159.0287 |
| 27 | 27.44 | Goshonoside-F4 | C32H54O12 | 665.3325[M-H]^-^ | 1.6 | 629.3620 |
|  |  |  |  | 648.3951[M+NH4]^+^ | -0.4 | 559.4874, 271.2422, 175.1485, 133.1020 |
| 28 | 28.53 | (16α)-16,17-dihydroxy-ent-kauran-2-one 17β-D- glucoside | C26H42O8 | 481.2811[M-H]^-^ | 0.8 | 319.2289, 301.2178 |
| 29 | 28.80 | Hyptatic acid | C30H48O6 | 503.3381[M-H]^-^ | 0.6 | 485.3295 |
| 30 | 28.80 | Sericic acid | C30H48O6 | 503.3381[M-H]^-^ | 0.6 | 485.3295 |
| 31 | 29.38 | 2α,19α,24-trihydroxyurs-12-ene-3-oxo-28-acid | C30H46O6 | 501.3222[M-H]^-^ | 0.0 | 457.2997, 453.3033 |
| 32 | 30.70 | 2α,19α-dihydroxy-3-oxo-12-ursen-28-oic acid | C30H46O5 | 487.3418[M+H]^+^ | -0.1 | 441.3375 |
|  |  |  | C30H46O5 | 485.3277[M-H]^-^ | 0.9 | 439.3249 |
| 33 | 33.96 | 2α-hydroxyursolic acid | C30H48O4 | 473.3627[M+H]^+^ | 0.3 | 409.3461, 313.2526, 207.1742, 205.1587, 203.1791, 189.1634, 177.1638, 175.1488 |
| 34 | 33.96 | Maslinic acid | C30H48O4 | 473.3627[M+H]^+^ | 0.3 | 409.3461, 313.2526, 207.1742, 205.1587, 203.1791, 189.1634, 177.1638, 175.1488 |

* Identified with authentic compounds.

**Table S2. Antibodies list.**

| **Name** | **Citation** | **Supplier** | **Cat no.** | **Dilution ratio** |
| --- | --- | --- | --- | --- |
| ZO-1 | IF | Proteintech | 21773-1-AP | 1:200 |
| Occludin | IF | Proteintech | 66378-1-Ig | 1:200 |
| Claudin-1 | IF | Proteintech | 28674-1-AP | 1:200 |
| CoraLite® Plus 488 | IF | Proteintech | RGAM002 | 1:400 |
| CoraLite® Plus 488 | IF | Proteintech | RGAR002 | 1:400 |
| CoraLite® Plus 594 | IF | Proteintech | RGAR004 | 1:400 |

**Table S3.** **Primers design for Real-Time PCR.**

| **Gene name** | **Forward (5’-3’)** | **Reverse (5’-3’)** |
| --- | --- | --- |
| *Paics* | ATGGCGACAGCCGTAGTAGT | CCTGGGGTGTCTAACAACTCA |
| *Nt5c2* | CCTCCTGGAGTGACCGCTTA | AACACCCGGTGATAGGCTTCT |
| *Atic* | GCCTCGTGGAATTTGCCAGA | AACCCTGTTAGCTCAGACACA |
| *Lacc1* | GTTCCAAAAGGAGAGATCCCAAA | TTAACACACCTACCGGAGTGA |
| *Ak3* | GTGTCGTCACGCATCACCAA | GCCAACACACCGATTTCTGT |
| *Tk2* | AGCAGTGGTTTGTATTGAGGG | ACATGAGGCTCAGAGGGTTATG |
| *Adss2* | ATGTCGATCTCCGAGAGCAG | GGCCAGCGTTATTCCCTCC |
| *Ada* | ACCCGCATTCAACAAACCCA | AGGGCGATGCCTCTCTTCT |
| *Pkm* | GCCGCCTGGACATTGACTC | CCATGAGAGAAATTCAGCCGAG |
| *Scd1* | TTCTTGCGATACACTCTGGTGC | CGGGATTGAATGTTCTTGTCGT |
| *Cd36* | ATGGGCTGTGATCGGAACTG | TTTGCCACGTCATCTGGGTTT |
| *Dgat2* | GCGCTACTTCCGAGACTACTT | GGGCCTTATGCCAGGAAACT |
| *Vldlr* | GAGTCTGACTTCGTGTGCAAA | GAACCGTCTTCGCAATCAGGA |
| *Il6* | TAGTCCTTCCTACCCCAATTTCC | TTGGTCCTTAGCCACTCCTTC |
| *Tnfa* | CAGGCGGTGCCTATGTCTC | CGATCACCCCGAAGTTCAGTAG |
| *Becn1* | ATGGAGGGGTCTAAGGCGTC | TGGGCTGTGGTAAGTAATGGA |
| *Pink1* | TTCTTCCGCCAGTCGGTAG | CTGCTTCTCCTCGATCAGCC |
| *Prkn* | GAGGTCCAGCAGTTAAACCCA | CACACTGAACTCGGAGCTTTC |
| *Fundc1* | AGCGATGACGAATCATACGAAG | CCACCCATTACAATCTGAGTAGC |
| *Bnip3* | CTGGGTAGAACTGCACTTCAG | GGAGCTACTTCGTCCAGATTCAT |
| *18s* | GTAACCCGTTGAACCCCATT | CCATCCAATCGGTAGTAGCG |
